# First-line tislelizumab plus chemotherapy versus placebo plus chemotherapy in adults with advanced or metastatic esophageal squamous cell carcinoma: a Japanese subgroup analysis of RATIONALE-306 with ≥ 3 years of follow-up

**DOI:** 10.1007/s10388-026-01199-y

**Published:** 2026-04-01

**Authors:** Takashi Ogata, Takashi Kojima, Ryu Ishihara, Hiroki Hara, Sebastian Yan, Sheng Xu, Ken Kato

**Affiliations:** 1 Department of Gastrointestinal Surgery, Kanagawa Cancer Center, Yokohama, Kanagawa Japan; 2 Department of Gastroenterology and Gastrointestinal Oncology, National Cancer Center Hospital East, Kashiwa-shi, Chiba Japan; 3 Department of Gastrointestinal Oncology, Osaka International Cancer Institute, Osaka, Japan; 4 Department of Gastroenterology, Saitama Cancer Center, Saitama, Japan; 5 Clinical Development, BeOne Medicines, Ltd., Beijing, China; 6 Statistics, BeOne Medicines, Ltd., Beijing, China; 7 Department of Head and Neck, Esophageal Medical Oncology, National Cancer Center Hospital, 5-1-1 Tsukiji, Chuo-ku, Tokyo, 104-0045 Japan

**Keywords:** Immunotherapy, Neoplasms, Drug therapy, Clinical trial, Japan

## Abstract

**Background:**

In the global phase 3 RATIONALE-306 study (NCT03783442), first-line tislelizumab plus chemotherapy showed significant overall survival (OS) benefit versus chemotherapy alone for unresectable locally advanced/metastatic esophageal squamous cell carcinoma (ESCC). We report post hoc results for the Japanese subgroup.

**Methods:**

Eligible Japanese patients were randomized (1:1) to tislelizumab 200 mg or placebo every 3 weeks plus chemotherapy (cisplatin plus fluoropyrimidine) and included in the Japanese analysis set. Endpoints included OS, progression-free survival (PFS), objective response rate (ORR), OS in patients with programmed death-ligand 1 (PD-L1) Tumor Area Positivity (TAP) score ≥ 10%, and safety.

**Results:**

Overall, 66/649 (10.2%) patients were randomized in Japan (n = 33 per arm). After a minimum follow-up of 37.9 months (data cutoff November 24, 2023), tislelizumab plus chemotherapy showed improvements in median OS versus placebo plus chemotherapy (24.5 vs. 15.1 months; hazard ratio [HR]: 0.75; 95% CI 0.43–1.30). An improvement in OS was also seen in patients with PD-L1 TAP score ≥ 10% (HR: 0.79; 95% CI 0.26–2.36). There was improvement in median PFS (HR: 0.77; 95% CI 0.45–1.32) and a higher ORR (63.6% vs. 45.5%) in the tislelizumab plus chemotherapy versus placebo plus chemotherapy arm, respectively. Treatment-related adverse events (TRAEs) with tislelizumab plus chemotherapy versus placebo plus chemotherapy occurred in, respectively, 45.5% versus 36.4% (any-grade) and 27.3% versus 6.1% (grade ≥ 3) of patients. No TRAE-related deaths occurred.

**Conclusions:**

After 3 years, first-line tislelizumab plus chemotherapy demonstrated sustained efficacy and a tolerable safety profile in Japanese patients with unresectable locally advanced/metastatic ESCC, consistent with the global RATIONALE-306 population.

**Supplementary Information:**

The online version contains supplementary material available at 10.1007/s10388-026-01199-y.

## Introduction

In Japan and many other Asian countries, esophageal squamous cell carcinoma (ESCC) is the predominant histological type of esophageal cancer [1, 2]. Recent advances in immunotherapy have revolutionized ESCC treatment. Monoclonal anti–programmed cell death protein-1 (PD-1) antibodies such as pembrolizumab, nivolumab, and tislelizumab, in combination with platinum-based chemotherapy, have shown superior survival outcomes compared with platinum-based chemotherapy as first-line treatment for ESCC [3–5].

Tislelizumab (BGB-A317) is a PD-1 inhibitor that has demonstrated high specificity and affinity and more complete blockade of PD-1/programmed death-ligand-1 (PD-L1) interaction compared with other PD-1 inhibitors [6]. Tislelizumab in combination with platinum-based chemotherapy is currently approved in Japan and several other countries/regions as first-line treatment for patients with unresectable locally advanced or metastatic ESCC [7–10].

The global, randomized, double-blind, phase 3 RATIONALE-306 study (NCT03783442) was pivotal in evaluating the efficacy of tislelizumab in combination with different investigator-chosen chemotherapy options as first-line treatment for unresectable locally advanced or metastatic ESCC. The primary analysis revealed that tislelizumab plus chemotherapy significantly improved overall survival (OS; stratified hazard ratio [HR]: 0.66 [95% confidence interval (CI) 0.54–0.80]) compared with placebo plus chemotherapy, with a manageable safety profile [5]. The improved OS was maintained at a minimum of 3 years of follow-up post study unblinding (stratified HR: 0.70 [95% CI 0.59–0.83]) [11].

We present a post hoc analysis of efficacy and safety/tolerability results from the Japanese subgroup of RATIONALE-306 at a minimum 3-year follow-up. The relationship between PD-L1 expression (using both combined positive score [CPS] and Tumor Area Positivity [TAP] score) and treatment outcomes was also explored in this subgroup.

## Materials and methods

### Study design and patients

Full details of the RATIONALE-306 study have been published [5], including the protocol and statistical analysis plan [5, 11]. For more details, see the Supplementary Methods, Online Resource.

The study adhered to International Council for Harmonisation Good Clinical Practice Guidelines, the Declaration of Helsinki, and local regulations. For each study site, independent ethics committee or institutional review board approvals were obtained. Written, informed consent was provided by all patients, and safety was monitored by an independent data-monitoring committee.

### Procedures and assessments

Patients were randomized 1:1 to 200 mg of tislelizumab or placebo combined with chemotherapy, intravenously, every 3 weeks on day 1 of each 21-day cycle. For the Japanese analysis set (JAS), chemotherapy was cisplatin 60–80 mg/m^2^ intravenously on day 1 in combination with a fluoropyrimidine (5-FU, 750–800 mg/m^2^ intravenously on days 1–5). Crossover between treatment arms was prohibited. Treatment continued until investigator-assessed disease progression per Response Evaluation Criteria in Solid Tumors version 1.1, unacceptable toxicity, death, or withdrawal of consent. For more details, see the Supplementary Methods, Online Resource.

### Endpoints

The following endpoints were assessed in the JAS: OS, investigator-assessed progression-free survival (PFS), objective response rate (ORR), duration of response (DoR), OS in the subgroups with PD-L1 TAP score cutoffs (≥ 10%, < 10%, ≥ 5%, < 5%, ≥ 1%, and < 1%) and CPS cutoffs (≥ 10, < 10, ≥ 5, < 5, ≥ 1, and < 1), and safety. Concordance of TAP score and CPS was also investigated (see the Supplementary Methods, Online Resource for details).

### Statistical analysis

This analysis focuses specifically on the JAS, which included all patients enrolled and randomized in RATIONALE-306 in Japan.The safety set comprised patients who received ≥ 1 dose of study treatment; all patients in the JAS received ≥ 1 dose of treatment and were therefore included in the safety set. For details on the post hoc analysis, see the Supplementary Methods, Online Resource.

## Results

### Patient disposition, demographic and baseline characteristics

Of the 649 enrolled/randomized patients, 66 were Japanese, from 19 sites across Japan (tislelizumab plus chemotherapy arm, n = 33; placebo plus chemotherapy arm, n = 33; Supplementary Fig. 1, Online Resource). The disposition data for the Japanese patients is presented in Supplementary Fig. 1, Online Resource. While most demographic and baseline disease characteristics were generally balanced between treatment arms in the subpopulation (Table 1), there were some notable differences. In the tislelizumab plus chemotherapy arm, more patients had an Eastern Cooperative Oncology Group performance status (ECOG PS) of 0, fewer were never-smokers, and none had locally advanced disease, versus the placebo plus chemotherapy arm.

**Table 1:** Baseline demographic and clinical characteristics of Japanese patients from RATIONALE-306

	Tislelizumab plus chemotherapy (n = 33)	Placebo plus chemotherapy (n = 33)
Age, years		
Median (min, max)	65.0 (42.0, 78.0)	70.0 (47.0, 76.0)
< 65	16 (48.5)	11 (33.3)
≥ 65	17 (51.5)	22 (66.7)
Sex		
Male	29 (87.9)	30 (90.9)
Female	4 (12.1)	3 (9.1)
Median BMI, kg/m^2^ (IQR)	20.8 (19.0–22.4)	20.4 (18.1–21.4)
ECOG performance status		
0	27 (81.8)	24 (72.7)
1	6 (18.2)	9 (27.3)
Tobacco consumption		
Never	1 (3.0)	4 (12.1)
Former	29 (87.9)	27 (81.8)
Current	3 (9.1)	2 (6.1)
Disease status at study entry		
Locally advanced	0 (0.0)	2 (6.1)
Metastatic	33 (100.0)	31 (93.9)
Number of metastatic sites at study entry		
0	0 (0.0)	2 (6.1)
1	18 (54.5)	17 (51.5)
2	10 (30.3)	9 (27.3)
≥ 3	5 (15.2)	5 (15.2)
Histological type		
Squamous cell carcinoma	33 (100.0)	33 (100.0)
PD-L1 expression		
TAP score ≥ 10%	12 (36.4)	7 (21.2)
TAP score < 10%	17 (51.5)	21 (63.6)
TAP score ≥ 5%	17 (51.5)	16 (48.5)
TAP score < 5%	12 (36.4)	12 (36.4)
TAP score ≥ 1%	24 (72.7)	26 (78.8)
TAP score < 1%	5 (15.2)	2 (6.1)
TAP score unknown^a^	4 (12.1)	5 (15.2)
CPS ≥ 10	11 (33.3)	12 (36.4)
CPS < 10	17 (51.5)	16 (48.5)
CPS ≥ 5	16 (48.5)	18 (54.5)
CPS < 5	12 (36.4)	10 (30.3)
CPS ≥ 1	26 (78.8)	26 (78.8)
CPS < 1	2 (6.1)	2 (6.1)
CPS unknown	5 (15.2)	5 (15.2)

Values are n (%) unless otherwise noted

*BMI* body mass index, *CPS* combined positive score, *ECOG* Eastern Cooperative Oncology Group, *IQR* interquartile range, *PD-L1* programmed death-ligand 1, *TAP* Tumor Area Positivity

^a^Unknown refers to patients without sample collection, with nonevaluable samples, or with scored unqualified samples

### Study drug exposure

At the time of study data cutoff (November 24, 2023), the minimum follow-up was 37.9 months. Median study treatment exposure time was longer with tislelizumab plus chemotherapy (7.0 months; interquartile range [IQR]: 2.5–8.1) versus the placebo plus chemotherapy arm (4.7 months; IQR: 2.7–6.9), respectively. Exposure to cisplatin plus fluoropyrimidine among patients in the JAS who received ≥ 1 dose of study treatment was generally similar, although two patients in the tislelizumab plus chemotherapy arm received 5-FU for more than 36 months, while the median treatment duration was less than 5 months (range: 4.1–4.9). In the tislelizumab plus chemotherapy and placebo plus chemotherapy arms, respectively, 78.8% and 84.8% received any subsequent anticancer systemic therapy and 45.5% and 63.6% received subsequent immunotherapy (Supplementary Table 1, Online Resource).

### Survival outcomes

**Fig. 1:**
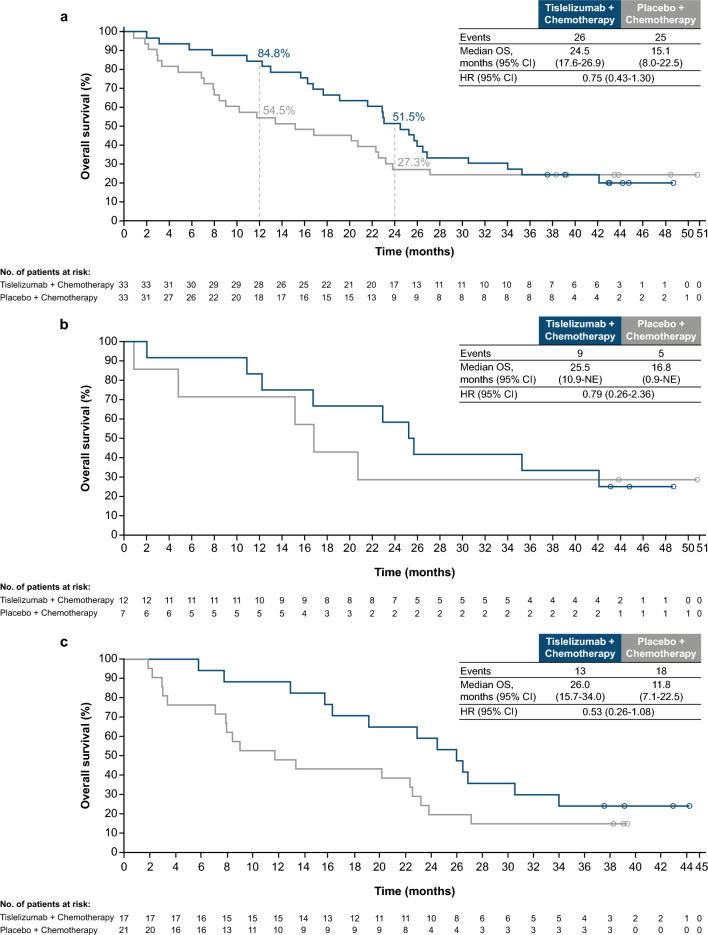
Kaplan–Meier plots of overall survival in the JAS (**a**) and in the JAS with tumor PD-L1 TAP score ≥ 10% (**b**) and < 10% (**c**). The HR for tislelizumab plus chemotherapy versus placebo plus chemotherapy was based on an unstratified Cox regression model only including treatment as a covariate. *CI* confidence interval, *HR* hazard ratio, *JAS* Japanese analysis set, *OS* overall survival, *PD-L1* programmed death-ligand 1, *TAP* Tumor Area Positivity

Median follow-up was 43.1 months (95% CI 37.6–44.8) and 41.4 months (38.3–48.5) in the tislelizumab plus chemotherapy and placebo plus chemotherapy arms, respectively. A longer median OS was reported with tislelizumab plus chemotherapy versus placebo plus chemotherapy (median OS: 24.5 months [95% CI 17.6–26.9] vs. 15.1 months [95% CI 8.0–22.5]; unstratified HR: 0.75 [95% CI 0.43–1.30]) (Fig. 1a). Higher OS rates were observed in the tislelizumab plus chemotherapy arm than the placebo plus chemotherapy arm at both 12 months (84.8% [95% CI 67.4–93.4] vs. 54.5% [95% CI 36.3–69.6]) and 24 months (51.5% [95% CI 33.5–66.9] vs. 27.3% [95% CI 13.6–42.9]) (Fig. 1a).

Improvement in OS with tislelizumab plus chemotherapy was observed in multiple PD-L1 expression subgroups. Median OS in the tislelizumab plus chemotherapy versus placebo plus chemotherapy arm in patients with a tumor PD-L1 TAP score ≥ 10% was 25.5 months (95% CI 10.9–not estimable [NE]) versus 16.8 months (95% CI 0.9–NE), respectively (unstratified HR: 0.79 [95% CI 0.26–2.36]; Fig. 1b).

In post hoc exploratory analyses of other PD-L1 TAP score subgroups, among patients with a PD-L1 TAP score < 10%, the median OS was 26.0 months (95% CI 15.7–34.0) versus 11.8 months (95% CI 7.1–22.5) in the tislelizumab plus chemotherapy arm versus the placebo plus chemotherapy arm, respectively (unstratified HR: 0.53 [95% CI 0.26–1.08]) (Fig. 1c). In patients with PD-L1 TAP scores ≥ 5% and < 5%, the median OS in the tislelizumab plus chemotherapy versus placebo plus chemotherapy arm was 26.0 (95% CI 16.8-NE) versus 13.5 (3.4–23.8) months and 23.7 (7.8–34.0) versus 16.8 (3.0–23.2) months, respectively (unstratified HR for TAP score ≥ 5%: 0.57 [95% CI 0.26–1.29], and for TAP score < 5%, 0.57 [0.24–1.35]). For TAP scores ≥ 1% and < 1%, median OS in the tislelizumab plus chemotherapy versus placebo plus chemotherapy arm was 26.2 (95% CI 22.9–42.1) versus 12.6 (7.1–22.3) months, and 15.7 (95% CI 5.8–NE) versus 23.6 (20.1–NE) months, respectively (unstratified HR for TAP score ≥ 1%: 0.47 [95% CI 0.25–0.90], and for TAP score < 1%: 4.40 [0.49–39.40]).

### Progression-free survival

**Fig. 2:**
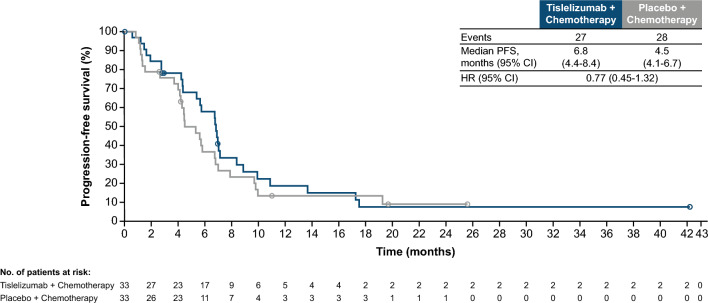
Kaplan–Meier plot of progression-free survival by investigator assessment in the JAS. The HR (tislelizumab plus chemotherapy vs. placebo plus chemotherapy) was based on an unstratified Cox regression model only including treatment as a covariate. *CI* confidence interval, *HR* hazard ratio, *JAS* Japanese analysis set

Patients treated with tislelizumab plus chemotherapy experienced a numerical improvement in investigator-assessed PFS versus those treated with placebo plus chemotherapy. Median investigator-assessed PFS was 6.8 months (95% CI 4.4–8.4) versus 4.5 months (95% CI 4.1–6.7) in the tislelizumab plus chemotherapy versus placebo plus chemotherapy arm, respectively (unstratified HR: 0.77 [95% CI 0.45–1.32]) (Fig. 2).

The 12-month PFS rate was 18.5% (95% CI 6.9–34.6) versus 13.3% (95% CI 4.2–27.6) in the tislelizumab plus chemotherapy versus placebo plus chemotherapy arm, respectively, and the 24-month PFS rates were 7.4% (95% CI 1.3–20.9) and 8.8% (95% CI 1.8–23.0), respectively (Fig. 2).

### Response outcomes

The ORR was 63.6% (21/33) versus 45.5% (15/33) for patients treated with tislelizumab plus chemotherapy versus placebo plus chemotherapy (odds ratio: 2.20 [95% CI 0.79–6.11]; *P* = 0.133). A complete response was observed in 12.1% (4/33) versus 3.0% (1/33) of patients in the tislelizumab plus chemotherapy arm versus the placebo plus chemotherapy arm, respectively (Table 2). Investigator-assessed median DoR with tislelizumab plus chemotherapy was 5.3 (95% CI 4.2–8.5) months versus 4.4 (95% CI 2.4–8.4) months with placebo plus chemotherapy (Supplementary Fig. 2, Online Resource).

**Table 2:** Response by investigator (JAS)

	Tislelizumab plus chemotherapy (n = 33)	Placebo plus chemotherapy (n = 33)
ORR, No	21	15
% (95% CI)^a^	63.6 (45.1–79.6)	45.5 (28.1–63.6)
Odds ratio for ORR (95% CI)^b^	2.20 (0.79–6.11)	
Complete response	4 (12.1)	1 (3.0)
Partial response	17 (51.5)	14 (42.4)
Stable disease^c^	7 (21.2)	11 (33.3)
Progressive disease	4 (12.1)	6 (18.2)
Not evaluable^d^	0 (0.0)	0 (0.0)
Not assessable^e^	1 (3.0)	1 (3.0)

Values are n (%) unless otherwise noted

*CI* confidence interval, *IRT* interactive response technology, *JAS* Japanese analysis set, *ORR* objective response rate

^a^Two-sided 95% CI was calculated using Clopper–Pearson method

^b^ORR, ORR differences, and odds ratios between groups were calculated using the Cochran–Mantel–Haenszel method, stratified by prior definitive therapy (yes vs. no) per IRT and investigator choice of chemotherapy option per IRT

^c^Stable disease includes stable disease and noncomplete response/nonprogressive disease

^d^Not evaluable is based on Response Evaluation Criteria in Solid Tumors version 1.1

^e^Patients with no postbaseline tumor assessment by the data cutoff, including those who discontinued the study (any reason) or died without having any postbaseline tumor assessment

### Concordance for TAP score and CPS in the JAS

Prevalence rates for PD-L1 expression were balanced between treatment arms and were comparable across arms by TAP score or CPS at different thresholds in the JAS. The results of the agreement analysis and Cohen’s k between TAP score and CPS are presented in Supplementary Fig. 3, Online Resource. Substantial agreement between TAP score and CPS was observed across the cutoffs at ≥ 1% versus ≥ 1 and ≥ 10% versus ≥ 10 in terms of overall percent agreement (OPA; 0.95 and 0.82, respectively) and Cohen’s k (0.70 and 0.62, respectively), and moderate agreement was observed at ≥ 5% versus ≥ 5 (OPA: 0.77; Cohen’s k: 0.52).

### Safety and tolerability

All patients reported having ≥ 1 treatment-emergent adverse event (TEAE) (Table 3). Grade ≥ 3 TEAEs were observed in 75.8% (25/33) of patients in each arm; the most common were decreased neutrophil count (24.2% vs. 36.4%) and anemia (21.2% vs. 6.1%) in the tislelizumab plus chemotherapy versus placebo plus chemotherapy arm, respectively. Serious TEAEs occurred in 45.5% (15/33) versus 30.3% (10/33) of patients in the tislelizumab plus chemotherapy versus placebo plus chemotherapy arm, respectively.

**Table 3:** TEAEs occurring in ≥ 10% of the JAS

	Tislelizumab plus chemotherapy (n = 33)		Placebo plus chemotherapy (n = 33)	
	All grades	Grade ≥ 3	All grades	Grade ≥ 3
Patients with ≥ 1 TEAE	33 (100.0)	25 (75.8)	33 (100.0)	25 (75.8)
Constipation	22 (66.7)	0 (0.0)	24 (72.7)	0 (0.0)
Stomatitis	18 (54.5)	0 (0.0)	18 (54.5)	0 (0.0)
Decreased appetite	17 (51.5)	3 (9.1)	17 (51.5)	3 (9.1)
White blood cell count decreased	17 (51.5)	2 (6.1)	19 (57.6)	4 (12.1)
Neutrophil count decreased	15 (45.5)	8 (24.2)	20 (60.6)	12 (36.4)
Nausea	14 (42.4)	1 (3.0)	17 (51.5)	1 (3.0)
Anemia	12 (36.4)	7 (21.2)	7 (21.2)	2 (6.1)
Diarrhea	9 (27.3)	2 (6.1)	10 (30.3)	0 (0.0)
Hiccups	9 (27.3)	0 (0.0)	10 (30.3)	0 (0.0)
Pyrexia	9 (27.3)	0 (0.0)	6 (18.2)	0 (0.0)
Infusion site extravasation	8 (24.2)	0 (0.0)	6 (18.2)	0 (0.0)
Peripheral sensory neuropathy	8 (24.2)	0 (0.0)	6 (18.2)	0 (0.0)
Hyponatremia	7 (21.2)	5 (15.2)	1 (3.0)	1 (3.0)
Hyperkalemia	6 (18.2)	2 (6.1)	1 (3.0)	0 (0.0)
Dysgeusia	6 (18.2)	0 (0.0)	8 (24.2)	0 (0.0)
Malaise	6 (18.2)	0 (0.0)	8 (24.2)	0 (0.0)
Platelet count decreased	5 (15.2)	0 (0.0)	5 (15.2)	0 (0.0)
Pneumonia	5 (15.2)	0 (0.0)	1 (3.0)	0 (0.0)
Vasculitis	5 (15.2)	0 (0.0)	1 (3.0)	0 (0.0)
Fatigue	4 (12.1)	1 (3.0)	8 (24.2)	0 (0.0)
Lymphocyte count decreased	4 (12.1)	1 (3.0)	4 (12.1)	0 (0.0)
Insomnia	4 (12.1)	0 (0.0)	11 (33.3)	0 (0.0)
Pneumonia aspiration	4 (12.1)	0 (0.0)	4 (12.1)	1 (3.0)
Alopecia	4 (12.1)	0 (0.0)	3 (9.1)	0 (0.0)
Pruritus	4 (12.1)	0 (0.0)	2 (6.1)	0 (0.0)
Generalized edema	3 (9.1)	0 (0.0)	4 (12.1)	0 (0.0)
Lipase increased	2 (6.1)	2 (6.1)	4 (12.1)	3 (9.1)
Renal impairment	2 (6.1)	1 (3.0)	5 (15.2)	0 (0.0)
Headache	2 (6.1)	0 (0.0)	5 (15.2)	0 (0.0)
Phlebitis	2 (6.1)	0 (0.0)	4 (12.1)	0 (0.0)
Weight decreased	2 (6.1)	0 (0.0)	5 (15.2)	0 (0.0)
Amylase increased	1 (3.0)	1 (3.0)	4 (12.1)	0 (0.0)
Edema peripheral	1 (3.0)	0 (0.0)	6 (18.2)	0 (0.0)

Values are n (%)

TEAE data presented include those that occurred in ≥ 10% of patients in either treatment group in patients who received at least one dose of study treatment. Patients with multiple events for a given preferred term are counted only once at the worst severity for the preferred term. Adverse event grades are evaluated based on National Cancer Institute-Common Terminology Criteria for Adverse Events version 4.03. Adverse events terms were coded using the Medical Dictionary for Regulatory Activities version 24.0. Adverse events are sorted by decreasing frequency of preferred term in the tislelizumab plus chemotherapy all-grades column then grade ≥ 3, then placebo plus chemotherapy all-grades then grade ≥ 3

*JAS* Japanese analysis set, *TEAE* treatment-emergent adverse event

Dose modification of tislelizumab/placebo due to TEAEs occurred in 69.7% (23/33) versus 72.7% (24/33) of patients in the tislelizumab plus chemotherapy arm versus the placebo plus chemotherapy arm, respectively. All but one of these dose modifications were dose delays. For one patient in the tislelizumab plus chemotherapy arm, the modification was a drug interruption. The most common TEAEs leading to dose modification in the tislelizumab plus chemotherapy arm versus the placebo plus chemotherapy arm were decreased neutrophil count (30.3% [10/33] vs. 33.3% [11/33]) and infusion site extravasation (21.2% [7/33] and 12.1% [4/33]). TEAEs leading to any treatment discontinuation occurred in 21.2% (7/33) of patients in the tislelizumab plus chemotherapy arm and 18.2% (6/33) in the placebo plus chemotherapy arm. Discontinuation of study treatment (tislelizumab plus chemotherapy/placebo plus chemotherapy) due to TEAEs was reported in 3.0% (1/33) versus 6.1% (2/33) of patients, respectively.

There were no TEAEs leading to death in either treatment arm. A summary of treatment-related adverse event (TRAEs) is included in Supplementary Tables 2 and 3, Online Resource.

Immune-mediated adverse events (imAEs) were reported in 39.4% (13/33) versus 15.2% (5/33) of patients in the tislelizumab plus chemotherapy arm versus the placebo plus chemotherapy arm, respectively. Of these, 12.1% (4/33) versus 0% were grade ≥ 3 and 15.2% (5/33) versus 6.1% (2/33) were considered serious imAEs in the tislelizumab plus chemotherapy versus placebo plus chemotherapy arm, respectively. As such, most potential imAEs were low grade (grade 1–2) and only led to treatment discontinuation in one patient in the tislelizumab plus chemotherapy arm.

## Discussion

With a minimum 3-year follow-up, this analysis of the RATIONALE-306 JAS, which accounted for ~ 10% of the global ITT population, revealed longer median OS and PFS in Japanese patients with unresectable locally advanced or metastatic ESCC who were treated with first-line tislelizumab plus chemotherapy versus placebo plus chemotherapy. Improvements in OS were observed in patients with PD-L1 TAP scores of ≥ 10%, < 10%, ≥ 5%, < 5%, and ≥ 1%, with similar median OS in patients treated with tislelizumab plus chemotherapy across these subgroups, although these results should be interpreted with caution due to limited sample size.

However, in Japanese patients with PD-L1 TAP score < 1%, median OS favored placebo plus chemotherapy (unstratified HR: 4.40; 95% CI 0.49–39.40), representing a reversal in the direction of the HR observed at higher PD-L1 cutoffs. Given the very small sample size of patients with PD-L1 TAP score < 1% (tislelizumab plus chemotherapy, n = 5; placebo plus chemotherapy, n = 2), wide CIs, and post hoc and exploratory nature of these analyses, the findings in the PD-L1–low subgroups should be interpreted with caution.

Despite some differences in baseline characteristics between the global ITT and JAS populations, the efficacy results in the JAS aligned closely with those of the global population [11]. Median OS with tislelizumab plus chemotherapy was 24.5 months versus 15.1 months with placebo plus chemotherapy (unstratified HR: 0.75 [95% CI 0.43–1.30]). Although not statistically significant, the observed OS improvement in the RATIONALE-306 JAS subgroup was consistent with the improved OS in the global population (stratified HR: 0.70 [95% CI 0.59–0.83]) [11]. It is important to note that cross-trial comparisons should be considered with caution due to inherent differences in study design and patient populations; however, these results were consistent with the OS benefit and HR observed in the KEYNOTE-590 trial of Japanese patients with treatment-naive ESCC (median OS: 17.6 months in the pembrolizumab plus chemotherapy arm vs. 11.7 months in the chemotherapy arm; HR: 0.71 [95% CI 0.47–1.09]) [12]. Both RATIONALE-306 and KEYNOTE-590 were randomized, double-blind, placebo-controlled phase 3 trials evaluating an anti-PD-1 antibody plus chemotherapy (cisplatin plus fluoropyrimidine) versus placebo plus chemotherapy in patients with advanced/metastatic ESCC. In both Japanese subgroups, the chemotherapy backbone was cisplatin plus fluoropyrimidine with minor dosing differences. RATIONALE-306 allowed cisplatin 60–80 mg/m^2^ and 5-FU 750–800 mg/m^2^, while KEYNOTE-590 used cisplatin 80 mg/m^2^ and 5-FU 800 mg/m^2^/day. Stratification factors differed between trials, with RATIONALE-306 stratifying by chemotherapy choice, region, and prior definitive therapy, and KEYNOTE-590 by geographic region, histology, and ECOG performance status. Baseline characteristics of the RATIONALE-306 Japanese subgroup showed a higher proportion of patients with ECOG PS 0 (77.3% vs. 71.6% in KEYNOTE-590 Japanese subgroup) and metastatic disease (97.0% vs. 89.4%). The RATIONALE-306 Japanese subgroup analysis was post hoc with a smaller sample size (n = 66) compared with the prespecified KEYNOTE-590 Japanese subgroup analysis (n = 141) [12].

While the PFS benefit with tislelizumab plus chemotherapy was more pronounced in the ITT population of the current study (HR: 0.60 [95% CI 0.50–0.72]) [11], the JAS also showed a trend toward improvement (HR: 0.80 [95% CI 0.47–1.36]), with smaller sample size (N = 66) likely contributing to the wider CI. In RATIONALE-306, median PFS in the JAS was 6.8 versus 4.5 months for tislelizumab plus chemotherapy versus chemotherapy, respectively. Although cross-trial comparisons have inherent limitations, as noted earlier, the PFS outcomes observed in the RATIONALE-306 Japanese patients were numerically higher than those reported in KEYNOTE-590, in which median PFS in Japanese patients was 6.3 versus 6.0 months in the pembrolizumab plus chemotherapy arm versus chemotherapy arm, respectively [12].

The antitumor activity of tislelizumab plus chemotherapy was evident in the JAS, with a higher ORR of 63.6% versus 45.5% for placebo plus chemotherapy, similar to the global population results (63.5% vs. 42.4%) [13]. The DoR was also longer in the tislelizumab plus chemotherapy arm, further supporting the clinical benefit of this combination in the JAS. Similarly, in the Japanese subgroup in KEYNOTE-590, the ORR with pembrolizumab plus chemotherapy was 56.8% and, with chemotherapy, 38.8% [12].

The safety/tolerability profile observed in the JAS was manageable and generally consistent with that in the global population. As observed in the global population, there was a higher incidence of serious TRAEs in the tislelizumab plus chemotherapy arm versus the placebo plus chemotherapy arm, though the proportions of serious TRAEs in the JAS and global populations were similar, at 24.2% and 19.8%, respectively [14]. The rate of grade ≥ 3 TRAEs appeared slightly lower in the JAS than the global population, particularly in the placebo plus chemotherapy arm, where grade ≥ 3 TRAEs were reported in 6.1% of the JAS compared with 20.2% of patients in the global population [14]. These differences may be due to the chemotherapy backbones used; paclitaxel plus platinum regimens were not used in the JAS. While there were five (1.5%) TRAEs leading to death in the tislelizumab plus chemotherapy arm and two (0.6%) TRAEs leading to death in the placebo plus chemotherapy arm in the global population, there were no TRAEs leading to death in the JAS [14]. Overall, the safety profile of tislelizumab among Japanese patients was comparable to that in the global population.

The analysis of PD-L1 expression using both TAP score and CPS revealed substantial agreement between these methods at various cutoffs, as demonstrated by the post hoc analysis conducted here in the JAS. The results showed moderate to substantial agreement in terms of OPA and Cohen’s k between the two scoring methods at multiple thresholds (1%, 5%, and 10%), suggesting potential interchangeability. This analysis was consistent with similar analyses in the global population, which showed substantial to almost perfect strength of agreement in terms of OPA (0.85–0.97) and Cohen’s k (0.67–0.85) at these cutoffs [13], and with an analysis of patients from the RATIONALE-302, RATIONALE-305, and RATIONALE-306 studies, which found similarly high agreement in OPA (0.82–0.97) and Cohen’s k (0.64–0.85) between TAP score and CPS at 1%, 5%, and 10% thresholds [15]. This finding is particularly important given that different regulatory agencies have adopted varying PD-L1 expression cutoffs, using various methods to measure PD-L1 expression for approval of immunotherapy in first-line ESCC [16–19]. For instance, for first-line treatment of patients with ESCC, the US Food and Drug Administration has approved nivolumab plus chemotherapy or ipilimumab for patients with PD-L1 ≥ 1 (measurement method not specified) and pembrolizumab plus chemotherapy for patients with CPS ≥ 1, while the European Medicines Agency has approved nivolumab plus chemotherapy or ipilimumab for patients with tumor cell PD-L1 expression ≥ 1% and pembrolizumab plus chemotherapy for patients with CPS ≥ 10 [16–19]. These differences in approval criteria and the use of different PD-L1 expression cutoffs for treatment approvals in various regions could potentially lead to disparities in patient access to immunotherapy and impact survival outcomes. As such, there is a pressing need for harmonization of PD-L1 testing and cutoff criteria across different regions and indications. While the September 26, 2024, meeting of the FDA’s Oncologic Drugs Advisory Committee harmonized on the equivalency of CPS versus TAP score PD-L1 measurements [20], standardization of PD-L1 assessment methods and cutoffs could help minimize disparities, ensuring more consistent access to effective treatments for patients with ESCC, and ultimately leading to more equitable healthcare outcomes. Japan is uniquely positioned to enhance the timely delivery of immunotherapy for patients with ESCC. With tislelizumab approved regardless of PD-L1 status and demonstrating efficacy across multiple PD-L1 expression levels, the decision to conduct PD-L1 testing prior to treatment initiation rests with individual physicians. In this context, the challenge of establishing a PD-L1 testing paradigm is comparatively less significant than in many other regions globally.

## Limitations

As is the nature of subgroup analyses, this post hoc analysis was restricted to a small, relatively homogeneous subpopulation of the global study, and no adjustment was performed for multiple comparisons. The exploratory PD-L1 subgroup analyses, particularly those with very small sample sizes such as the PD-L1 TAP score < 1% subgroup, should be interpreted with caution given the post hoc nature and limited statistical power of these analyses. Additionally, some of the baseline characteristics within the JAS (ECOG PS, smoking status, patients with locally advanced disease) were less balanced between arms. While these differences were small, potential bias toward poorer outcomes in the placebo plus chemotherapy arm in this subgroup cannot be ruled out. Compared with the global population, the JAS had more patients with ECOG PS of 0 (77.3% vs. 32.8%) and metastatic disease (97.0% vs. 86.4%) [5]. Additionally, more patients in the JAS received subsequent systemic therapy (81.8%) compared with the global population (54.7%) across both treatment groups [14]; this was expected due to the higher proportion of patients with ECOG PS of 0. The higher proportion of patients in the JAS with metastatic disease may reflect practice patterns in Japan, where surgery is more likely to be performed for locally advanced disease compared with other countries [21, 22].

## Conclusions

This 3-year follow-up subgroup analysis from RATIONALE-306 provides compelling evidence for the efficacy and safety/tolerability of tislelizumab plus chemotherapy as a first-line treatment for Japanese patients with unresectable locally advanced or metastatic ESCC, consistent with earlier data cutoffs. The consistency of these results with the global population data, the durability of the treatment effect observed, and the long-term manageable safety profile of tislelizumab plus chemotherapy further support the use of this combination in clinical practice.

## Data sharing

On request, and subject to certain criteria, conditions, and exceptions, BeOne Medicines, Ltd. will provide access to individual de-identified participant data from applicable BeOne Medicines-sponsored studies. BeOne Medicines shares data only when permitted by applicable data privacy and security laws and regulations, shares when it is feasible to do so without compromising the privacy of the study participants, and other considerations. Data requests may be submitted to ClinicalTrials@beonemed.com.

## Supplementary Information

Below is the link to the electronic supplementary material.

Supplementary file1 (DOCX 386 kb)

## Declarations

### Meeting presentation

Data were presented in part at the American Society of Clinical Oncology Gastrointestinal Cancers Symposium, San Francisco, CA, USA, January 23–25, 2025.
