# Supplementary material for: First-line tislelizumab plus chemotherapy versus placebo plus chemotherapy in adults with advanced or metastatic esophageal squamous cell carcinoma: a Japanese subgroup analysis of RATIONALE-306 with ≥ 3 years of follow-up
Source: Esophagus. 2026 Apr 1;23(3):501–11. doi: 10.1007/s10388-026-01199-y (PMC13319636; doi:10.1007/s10388-026-01199-y)
Supplement: Supplementary file 1 — Supplementary file1 (DOCX 386 kb) [file 10388_2026_1199_MOESM1_ESM.docx]

# Online Resource

**First-line tislelizumab plus chemotherapy versus placebo plus chemotherapy in adults with advanced or metastatic esophageal squamous cell carcinoma: a Japanese subgroup analysis of RATIONALE-306 with ≥3 years of follow-up**

**Article in *Esophagus***

Takashi Ogata ꞏ Takashi Kojima ꞏ Ryu Ishihara ꞏ Hiroki Hara ꞏ Sebastian Yan ꞏ Sheng Xu ꞏ Ken Kato

Corresponding Author: Ken Kato, Department of Head and Neck, Esophageal Medical Oncology, National Cancer Center Hospital, Tokyo, Japan

E-mail: [kenkato@ncc.go.jp](mailto:kenkato@ncc.go.jp)

## Supplementary Methods

### Study design and patients

Briefly, adult patients (≥18 years) with histologically confirmed, unresectable, locally advanced or metastatic ESCC who received no prior systemic therapy for advanced disease and who were ineligible for definitive therapies (including surgery or radiation) according to the local investigator, were eligible to enroll. If patients previously received neoadjuvant or adjuvant therapy with platinum-based chemotherapy, they must have had at least 6 months treatment-free before trial initiation. Patients were enrolled regardless of PD-L1 expression.

### Procedures and assessments

Tumor expression of PD-L1 was centrally assessed retrospectively by TAP score, calculated as the total percentage of tumor area (tumor and any desmoplastic stroma) covered by tumor cells with PD-L1 membrane staining at any intensity and tumor-associated immune cells with PD-L1 staining at any intensity. TAP score was assessed by pathologists using the VENTANA PD-L1 (SP263) Assay (Roche Diagnostics, Indianapolis, IN, USA) [1]. Pathologists in the central laboratory then scored the same stained samples using CPS. CPS was calculated as the number of PD-L1–expressing tumor cells, lymphocytes, and macrophages divided by the total number of viable tumor cells, multiplied by 100 [2].

### Endpoints

Assessments included a post hoc subgroup analysis of OS using PD-L1 expression, defined by cutoffs of TAP scores (≥10%, <10%, ≥5%, <5%, ≥1%, and <1%) and CPS (≥10, <10, ≥5, <5, ≥1, and <1) [3]. Concordance (Cohen’s k) of TAP score and CPS at multiple cutoffs and interclass correlation coefficient were also investigated. The value of Cohen’s k was interpreted as k ≤ 0 = no agreement, 0.01 to 0.20 = slight agreement, 0.21 to 0.40 = fair agreement, 0.41 to 0.60 = moderate agreement, 0.61 to 0.80 = substantial agreement, and 0.81 to 1.00 = almost perfect agreement [4]. Intraclass correlation coefﬁcients of <0.5, 0.5 to 0.75, >0.75 to 0.90, and >0.90 were interpreted as poor, moderate, good, and excellent reliability, respectively [4,5].

### Statistical Analysis

For this post hoc analysis of the JAS, OS and PFS were estimated with a log-rank test stratified by factors used in randomization. HRs and two-sided 95% CIs were estimated with a Cox regression model including treatment as a covariate. Estimations of median OS (in the JAS and each PD-L1 TAP score subgroup) and PFS were conducted using the Kaplan-Meier method. For median OS and PFS, a generalized Brookmeyer and Crowley method was used to construct the two-sided 95% CIs. The Cochran-Mantel-Haenszel test, adjusting for prespecified stratification factors, was used to test ORR; the two-sided 95% CI for odds ratio was calculated along with Clopper-Pearson 95% CIs of ORR in each treatment group. Analyses at a minimum 3-year follow-up were descriptive, and descriptive statistics were also used to analyze safety data. SAS (SAS Institute, Cary, NC, USA) version 9.4 or higher was used for all analyses.

**Supplementary references**

1. Liu C, Fang F, Kong Y, et al. Tumor Area Positivity (TAP) score of programmed death-ligand 1 (PD-L1): a novel visual estimation method for combined tumor cell and immune cell scoring. Diagn Pathol 2023;18:48.

2. Paintal AS, Brockstein BE. PD-L1 CPS scoring accuracy in small biopsies and aspirate cell blocks from patients with head and neck squamous cell carcinoma. Head Neck Pathol 2020;14:657-65.

3. Raymond E, Xu J, Kato K, et al. Tislelizumab (TIS) + chemotherapy (CT) vs placebo (PBO) + CT in locally advanced unresectable or metastatic esophageal squamous cell carcinoma. PD-L1 biomarker analysis from RATIONALE-306. Presented at: European Society for Medical Oncology World Congress on Gastrointestinal Cancer; June 26-29, 2024, Munich, Germany.

4. McHugh ML. Interrater reliability: the kappa statistic. Biochem Med (Zagreb) 2012;22:276-82.

5. Koo TK, Li MY. A Guideline of selecting and reporting intraclass correlation coefficients for reliability research. J Chiropr Med 2016;15:155-63.

### Supplementary Table 1 Subsequent anticancer therapy (Japanese analysis set)

| Anticancer therapy, n (%) | Tislelizumab plus chemotherapy  (n = 33) | Placebo plus chemotherapy (n = 33) |
| --- | --- | --- |
| Any subsequent anticancer systemic therapy | 26 (78.8) | 28 (84.8) |
| Chemotherapy | 24 (72.7) | 20 (60.6) |
| Immunotherapy | 15 (45.5) | 21 (63.6) |
| Other | 6 (18.2) | 2 (6.1) |

### Supplementary Table 2 Summary of TRAEs in the JAS

| n (%) | Tislelizumab plus chemotherapy  (n = 33) | Placebo plus chemotherapy  (n = 33) |
| --- | --- | --- |
| Patients with ≥1 TRAE | 31 (93.9) | 32 (97.0) |
| Tislelizumab or placebo related | 16 (48.5) | 12 (36.4) |
| Chemotherapy related | 31 (93.9) | 32 (97.0) |
| TRAEs of grade ≥3 | 22 (66.7) | 19 (57.6) |
| Tislelizumab or placebo related | 9 (27.3) | 2 (6.1) |
| Chemotherapy related | 18 (54.5) | 18 (54.5) |
| Serious TRAE | 12 (36.4) | 5 (15.2) |
| Tislelizumab or placebo related | 9 (27.3) | 1 (3.0) |
| Chemotherapy related | 5 (15.2) | 4 (12.1) |
| TRAEs leading to death | 0 (0.0) | 0 (0.0) |
| TRAEs leading to any treatment discontinuation | 7 (21.2) | 5 (15.2) |
| TRAEs leading to tislelizumab/placebo discontinuation | 1 (3.0) | 0 (0.0) |
| TRAEs leading to any chemotherapy component discontinuation | 7 (21.2) | 5 (15.2) |
| TRAEs leading to any dose modification^a^ | 25 (75.8) | 28 (84.8) |
| TRAEs leading to tislelizumab/placebo dose modification | 19 (57.6) | 21 (63.6) |
| Dose delay | 19 (57.6) | 21 (63.6) |
| TRAEs leading to any chemotherapy component dose modification | 25 (75.8) | 28 (84.8) |
| Dose delay | 19 (57.6) | 21 (63.6) |

Adverse event grades are evaluated based on National Cancer Institute-Common Terminology Criteria for Adverse Events version 4.03. Adverse events terms were coded using the Medical Dictionary for Regulatory Activities version 24.0

^a^The types of dose modification include dose delay, infusion interruption, infusion rate decreased and dose reduction for chemotherapy; dose delay, infusion interruption and infusion rate decreased for tislelizumab/placebo

*JAS* Japanese analysis set, *TRAE* treatment-related adverse event

### Supplementary Table 3 TRAEs occurring in ≥10% of the JAS

|  | Tislelizumab plus chemotherapy  (n = 33) | | Placebo plus  chemotherapy  (n = 33) | |
| --- | --- | --- | --- | --- |
|  | All grades | Grade ≥3 | All grades | Grade ≥3 |
| Patients with ≥1 TRAE | 31 (93.9) | 22 (66.7) | 32 (97.0) | 19 (57.6) |
| White blood cell count decreased | 17 (51.5) | 2 (6.1) | 19 (57.6) | 4 (12.1) |
| Stomatitis | 16 (48.5) | 0 (0.0) | 17 (51.5) | 0 (0.0) |
| Neutrophil count decreased | 15 (45.5) | 8 (24.2) | 19 (57.6) | 11 (33.3) |
| Decreased appetite | 14 (42.4) | 2 (6.1) | 17 (51.5) | 3 (9.1) |
| Nausea | 14 (42.4) | 1 (3.0) | 16 (48.5) | 1 (3.0) |
| Anemia | 12 (36.4) | 7 (21.2) | 7 (21.2) | 2 (6.1) |
| Constipation | 7 (21.2) | 0 (0.0) | 8 (24.2) | 0 (0.0) |
| Hiccups | 7 (21.2) | 0 (0.0) | 8 (24.2) | 0 (0.0) |
| Peripheral sensory neuropathy | 7 (21.2) | 0 (0.0) | 5 (15.2) | 0 (0.0) |
| Hyponatremia | 6 (18.2) | 5 (15.2) | 1 (3.0) | 1 (3.0) |
| Dysgeusia | 6 (18.2) | 0 (0.0) | 8 (24.2) | 0 (0.0) |
| Malaise | 6 (18.2) | 0 (0.0) | 8 (24.2) | 0 (0.0) |
| Diarrhea | 5 (15.2) | 1 (3.0) | 8 (24.2) | 0 (0.0) |
| Platelet count decreased | 5 (15.2) | 0 (0.0) | 5 (15.2) | 0 (0.0) |
| Vasculitis | 5 (15.2) | 0 (0.0) | 1 (3.0) | 0 (0.0) |
| Lymphocyte count decreased | 4 (12.1) | 1 (3.0) | 3 (9.1) | 0 (0.0) |
| Alopecia | 4 (12.1) | 0 (0.0) | 3 (9.1) | 0 (0.0) |
| Fatigue | 3 (9.1) | 1 (3.0) | 8 (24.2) | 0 (0.0) |
| Infusion site extravasation | 3 (9.1) | 0 (0.0) | 4 (12.1) | 0 (0.0) |
| Renal impairment | 2 (6.1) | 1 (3.0) | 5 (15.2) | 0 (0.0) |
| Weight decreased | 2 (6.1) | 0 (0.0) | 5 (15.2) | 0 (0.0) |
| Phlebitis | 2 (6.1) | 0 (0.0) | 4 (12.1) | 0 (0.0) |
| Amylase increased | 1 (3.0) | 0 (0.0) | 4 (12.1) | 0 (0.0) |

Values are n (%)

TRAE data presented include those that occurred in ≥10% of patients in either treatment group in patients who received at least one dose of study treatment. Patients with multiple events for a given preferred term are counted only once at the worst severity for the preferred term, respectively. Adverse event grades are evaluated based on National Cancer Institute-Common Terminology Criteria for Adverse Events version 4.03. Adverse events terms were coded using the Medical Dictionary for Regulatory Activities version 24.0. Adverse events are sorted by decreasing frequency of preferred term in the tislelizumab plus chemotherapy all grades then grade ≥3, then placebo plus chemotherapy all grades then grade ≥3

*JAS* Japanese analysis set, *TRAE* treatment-related adverse event

### Supplementary Fig. 1 Patient disposition (Japanese analysis set)


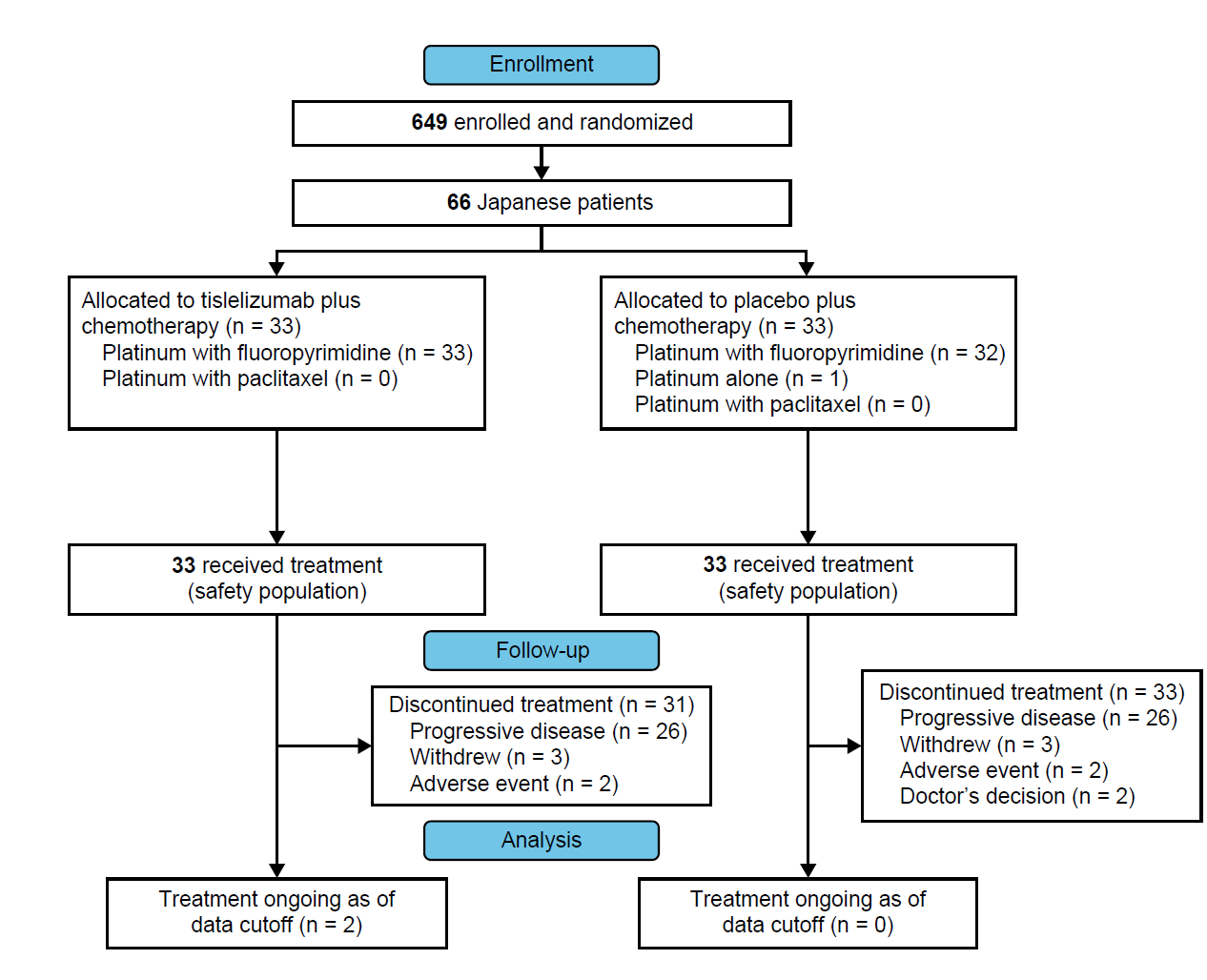


### Supplementary Fig. 2 Kaplan–Meier plot of duration of response by investigator assessment in the Japanese analysis set


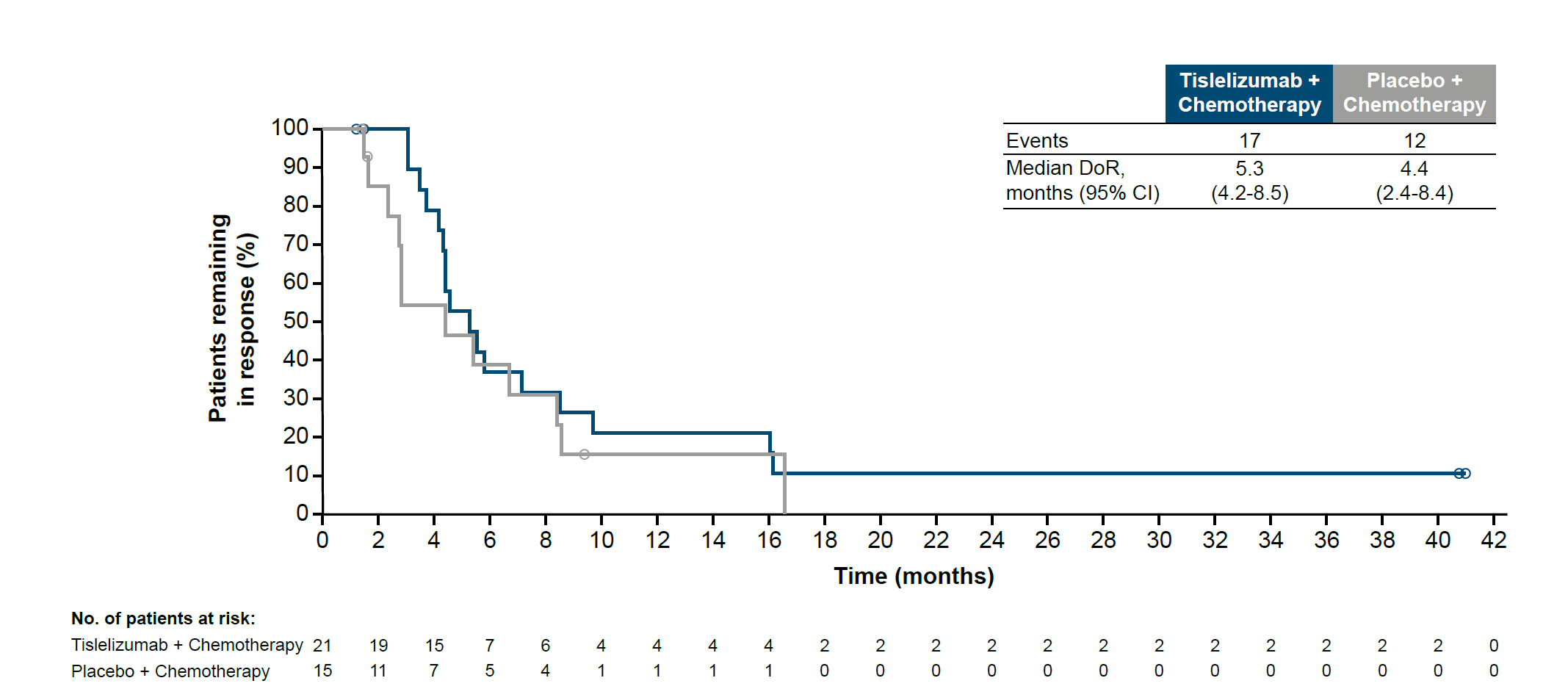


*CI* confidence interval, *DoR* duration of response

### Supplementary Fig. 3 Agreement analysis between TAP score and CPS (Japanese analysis set)


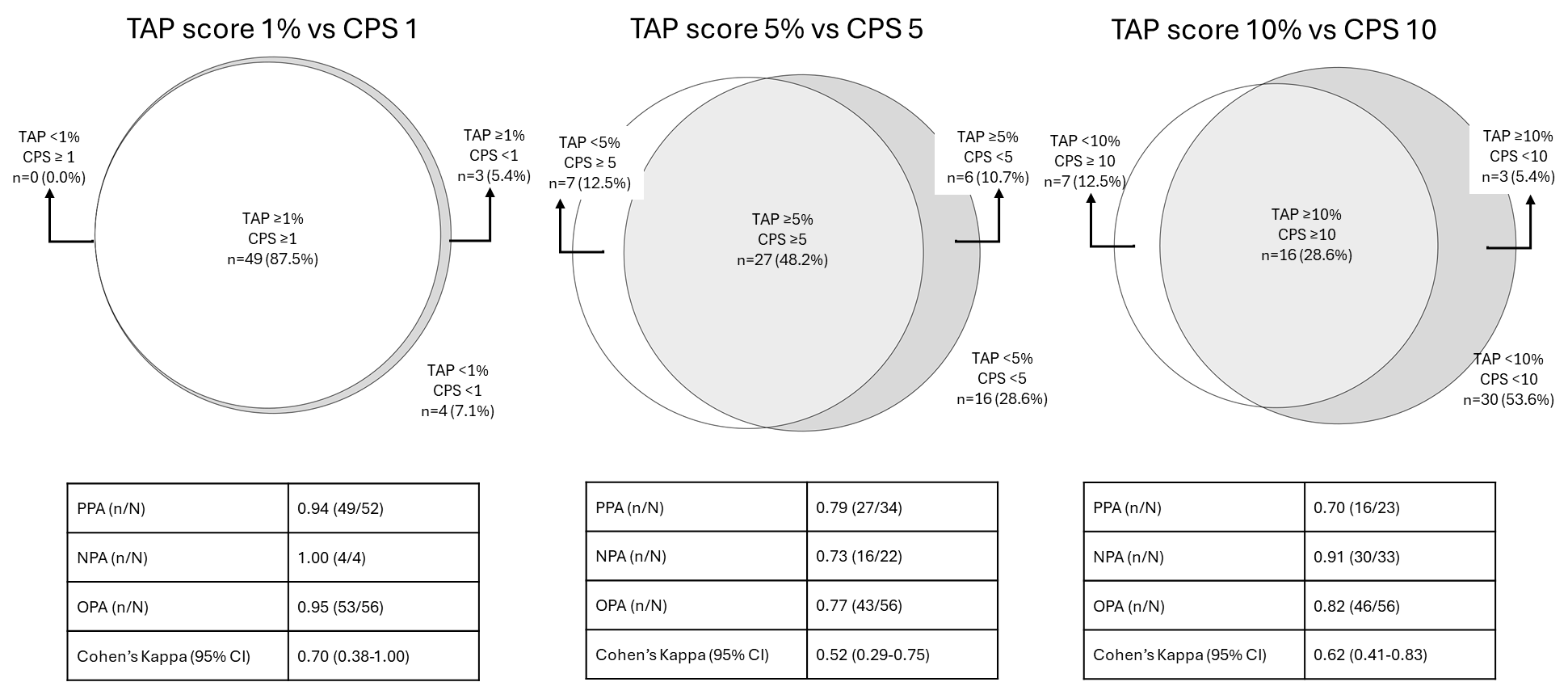


*CI* confidence interval, *CPS* combined positive score, *NPA* negative percent agreement, *OPA* overall percent agreement, *PPA* positive percent agreement, *TAP* Tumor Area Positivity
